# Barriers and enablers to achieving clinical procedure competency-based outcomes in a national paediatric training/residency program—a multi-centered qualitative study

**DOI:** 10.1186/s12909-023-04928-4

**Published:** 2023-12-13

**Authors:** Waqas Ullah Khan, John Twomey, Ethel Ryan, Therese Martin, Myeda Kamal, Pak Lok Boris Cheng, Clodagh O’Gorman, Dara Byrne

**Affiliations:** 1 Department of Psychiatry, School of Medicine, University of Limerick, Limerick, Ireland; 2 Department of Psychiatry, University Hospital Limerick, St Nessan’s Rd, Dooradoyle, County Limerick, Ireland; 3 Department of Paediatrics, School of Medicine, University of Limerick, Limerick, Ireland; 4 Department of Paediatrics, University Hospital Limerick, Limerick, Ireland; 5 Department of Paediatrics, School of Medicine, University of Galway, Galway, Ireland; 6 Department of Paediatrics, Galway University Hospital, Galway, Ireland; 7 Department of Biomedical Engineering, Trinity College Dublin, Dublin, Ireland; 8 Ballinasloe General Practice Specialist Training Scheme, Ballinasloe, Ireland; 9 School of Medicine, University of Galway, Galway, Ireland; 10 The Irish Centre for Applied Patient Safety and Simulation, School of Medicine, University of Galway, Galway, Ireland

**Keywords:** Competency-based Medical Education, Simulation-Based Medical Education, Paediatric Medicine, Training/Residency program, Qualitative study

## Abstract

**Background:**

In 2018, the Royal College of Physicians of Ireland revised its paediatric training program to a competency-based medical education (CBME) training/residency curriculum. This included a requirement to achieve competence in a number of core procedural skills to progress within the program. Internationally, simulation-based medical education (SBME) is gaining interest as an effective teaching pedagogy for training procedural skill competency. The objectives of this study were to (1) identify enablers and barriers for paediatric trainees to achieve their required procedural competencies, (2) gain insight on the feasibility of achieving the required procedural skills, and (3) explore what simulation-based resources are used as well as their role in achieving the required procedural skill competencies.

**Methods:**

A multi-centered qualitative study using semi-structured interviews was performed. Twenty-four paediatric consultants and trainees were recruited from two academic tertiary hospitals using purposive and snowball sampling. Interviews were conducted between March and September 2021, audio recorded, transcribed, and analyzed using thematic analysis.

**Results:**

Three main themes regarding enablers for achieving procedural competencies were reported and include having protected training time, routine assessments, and a standardized curriculum. Barriers to achieving procedural competencies focused mainly on limited clinical exposure. The use of SBME was recommended by all participants (n = 24, 100%) to assist in achieving procedural competencies and most (n = 15, 62.5%) reported it is feasible to attain the required procedural skills in the paediatric CBME program.

**Conclusion:**

It is feasible to achieve the required procedural competencies for most paediatric trainees, but this can be improved with protected training time, routine assessments, and a standardized curriculum. Barriers to achieving these skills mainly center on limited clinical exposure, which can be remedied by SBME. Further research is warranted to determine the costs and types of SBME tools available as well as teaching pedagogies to support paediatric trainees achieve their required procedural competencies.

## Introduction

In medicine, achieving a competency indicates a healthcare professional has demonstrated the ability to effectively perform a specific activity that integrates the required knowledge, skills, values, and attitudes [1]. As competencies are observed and demonstrated, they can be assessed to ensure a learner acquires them adequately. Similar to stacking blocks, competencies can be assembled and allow for progressive development [1]. For trainees with deficiencies in certain areas of knowledge, procedural skills, or professionalism, a competency-based training approach can provide an ‘‘early warning system’’ to guide remedial action [2].

Competency-based medical education (CBME) is a shift in clinical training that recognizes not all medical trainees learn the required skills at the same rate. More specifically, CBME programs address concerns regarding the varying abilities of medical trainees along with the risks and discrepancies they create in patient care [1–3]. In contrast to time-based medical training programs, which use summative assessments and allow trainees to progress after a certain duration, CBME programs require trainees satisfactorily demonstrate they possess specific competencies before advancing, promotes lifelong learning, and relies on continuous formative assessments that integrate feedback between the trainer and trainee [1–3].

The notion of a CBME medical training curriculum is not novel and in 1978, a World Health Organization (WHO) report stated “the intended output of a competency-based program is a health professional who can practice medicine at a defined level of proficiency, in accord with local conditions, to meet local needs” [4]. Competency-based medical education programs are becoming the predominant method to train healthcare professionals and have been approved by regulatory agencies in several countries. This includes the Accreditation Council for Graduate Medical Education (ACGME) in the United States of America (USA), the College of Family Physicians of Canada and Royal College of Physicians and Surgeons of Canada, as well as the accrediting or licensing bodies in Scotland, the Netherlands, and Australia [5].

Similar to CBME training programs, simulation-based medical education (SBME) has seen growing interest as an effective teaching pedagogy. Many healthcare institutions have simulation facilities and research shows an effective translation of skills learned in the lab to clinical practice [6, 7]. Through SBME, clinical knowledge as well as procedural, communication, leadership, and teamwork skills can also be learnt, measured, and certified in a safe and efficient manner [6, 7]. However, the perspectives of clinical trainers and trainees regarding the role of SBME tools to support the achievement of required procedural competencies in a CBME training program have not been explored.

In 2018, the Royal College of Physicians of Ireland (RCPI) revised their paediatric curriculum to a CBME program for basic specialist training (similar to a residency program) [8]. The objectives of this study are to examine the perspectives of paediatric consultants and trainees on the challenges of implementing and operationalizing the new curriculum. This was performed by identifying and exploring the enablers as well as barriers to achieving the required procedural skills in the paediatric CBME training program. Another aim was to gain insight on the feasibility to achieve the required procedural competencies in the paediatric CBME training program. Finally, this study sought to explore the role of SBME in supporting procedural skill attainment in the paediatric CBME training program.

## Methods

### Study design and participants

A qualitative study was performed using a hermeneutic phenomenological approach. This allows one to examine human experiences of participants (phenomenology) through the descriptions they provide as well as interpret these “lived experiences” (hermeneutics) [9]. The phenomenon under investigation was the implementation and operationalization of Ireland’s paediatric CBME training program through the achievement of procedural competencies. Questions asked were designed to obtain information on the perspectives of paediatric consultants and trainees regarding the RCPI’s CBME training program.

This was a multi-centered study with participants recruited from Galway University Hospital (GUH) and University Hospital Limerick (UHL) paediatric departments. Both hospitals are teaching centers affiliated with the University of Galway and the University of Limerick; two of Ireland’s largest universities and populated cities (Galway and Limerick). Recruitment was performed using purposive and snowball sampling to select a diverse population with respect to the number of years and level of experience working/training in paediatrics, medical education background, and gender. Paediatric consultants and trainees (both in and not in a structured RCPI program) with awareness of or participation in the CBME procedural skill training program were eligible. Participants who were not reachable by email after 2 attempts were excluded. The sample size was determined by data saturation, which occurs when no new themes regarding the experiences of study participants emerge. Participants had no previous interactions with the interviewer (WUK) who is a psychiatry trainee physician that has conducted qualitative and quantitative research studies in the fields of medical education and paediatrics.

Ethics approval for this study was received from the Clinical Institutional Research and Ethics Review Boards in GUH and UHL. The study objectives as well as its voluntary nature were explained to participants and both verbal and written informed consent were obtained prior to each interview. Confidentiality was obtained by removing all identifying information from the transcripts and assigning codenames to each participant in the data collection tools. This study followed the Standards for Reporting Qualitative Research guidelines [10].

### Procedures

One investigator (WUK) conducted three pilot interviews with two paediatric consultants (1 from GUH and 1 from UHL) and one paediatric higher specialist trainee (from UHL) in January 2021. For the pilot interviews, a preliminary version of the interview guide was developed by reviewing the paediatric CBME training program procedural skill requirements and using the expertise of four of the study investigators who are paediatric (ER, JT, and COG) and surgical training consultants (DB) in GUH or UHL. Using feedback from the pilot interviews, the interview guide was revised and a formal study interview outline was developed.

At the start of each semi-structured interview, the participant’s role in the hospital (e.g., paediatric consultant, higher specialist training/HST program trainee, basic specialist training/BST program trainee, and non-consultant hospital doctor/NCHD in a non-structured RCPI paediatric program), gender, university from which they received their medical degree, and number of years they practiced or trained in paediatrics were obtained. These questions were followed by a broad data-generating question: “In your opinion, what makes a CBME training program successful in providing trainees with the opportunities to achieve their required procedural skill-based competencies?” Open-ended follow-up questions were then asked to collect detailed descriptions. These questions are categorized as: (1) perspectives on enablers to implement the CBME curriculum, (2) perspectives and themes on the feasibility to achieve the required procedural competencies in the current paediatric CBME training program, (3) procedural competencies identified as difficult to achieve and themes concerning barriers to completing them, and (4) identifying simulation resources used and themes on whether there is a role for SBME tools to support trainees achieve their procedural competencies. Probing questions such as “please explain” or “tell me more about that” were used to encourage an in-depth discussion.

### Data collection

Semi-structured interviews were conducted individually and arranged at a time convenient for participants between March 2021 and September 2021. All interviews were conducted by one study investigator (WUK) either in-person or via telephone. At the start of each interview, the participant was informed there were no right or wrong answers and reassured that the information provided would not influence their training or career prospects. Interviews lasted between 13 and 35 min, with permission they were audio recorded and transcribed by one member of the research team (WUK) and reviewed for accuracy by two study investigators (MK and PLBC). The researcher conducting the interviews (WUK) also recorded handwritten notes on questionnaire forms throughout the interviewing process and data collection occurred simultaneously with data analysis.

### Data analysis

Haase’s eight-step qualitative analytic approach adapted from Colaizzi’s method was used to examine the transcribed interviews/transcripts [11, 12]. This method includes reading each transcript several times to obtain an understanding of meanings conveyed, identifying significant phrases and restating them in general terms, formulating meanings and validating them through discussions as well as consensus with the research team, identifying and organizing themes into clusters and categories, and developing full descriptions of themes. One investigator (WUK) met with co-investigators (MK, PLBC, and DB) throughout the analysis to discuss and refine the themes identified.

Several approaches were used to ensure validity in the data analysis. This included conducting in-depth interviews followed by peer debriefing to uncover research biases and assumptions. Two investigators (WUK and MK) also analyzed the transcripts independently by bracketing data on preconceived notions and following the adapted Colaizzi method described above. This helped mitigate potentially harmful effects of presumptions that can impact qualitative research analysis [13]. Regular meetings among the diverse group of study investigators with various professional backgrounds (paediatrics, psychiatry, surgery, medical and surgical simulation training, family medicine, and biomedical engineering) served the purpose of triangulation [14]. Finally, transferability was achieved by considering differences in participant characteristics, such as the level of paediatric work/training experience, gender, and work/training site.

## Results

### Participant characteristics

Participant characteristics are summarized in Table 1. Of the 24 participants interviewed in this study, 17 (70.8%) were female. Most paediatric clinicians and trainees obtained their medical degree from a university in Ireland (n = 19, 79.2%) with the remainder from universities in Africa (n = 2, 8.3%), Asia (n = 2, 8.3%), and Eastern Europe (n = 1, 4.2%). Purposive snowball sampling allowed for an almost even distribution of paediatric hospital professionals that included 7 (29.2%) consultants, 5 (20.8%) HST program trainees, 6 (25.0%) BST program trainees, and 6 (25.0%) NCHDs in a non-structured RCPI paediatric program. Similarly, there was an almost equal distribution of participants divided into subgroups according to their number of years working/training in paediatric medicine; 6 (25.0%) with ≤ 3years, 9 (37.5%) with 4 to 9 years, and 9 (37.5%) with ≥ 10 years. Those clinicians with ≤ 3years of work/training experience were all BST program trainees (n = 6, 25%), while 5 participants with 4 to 9 years of work/training experience were HST program trainees and the remaining 4 were paediatric NCHDs in a non-structured RCPI paediatric program. Concerning participants who had ≥ 10 years of work/training experience, 7 were paediatric consultants and 2 were NCHDs in a non-structured RCPI paediatric program.

**Table 1:** Participant Characteristics

Characteristic	Number (%) of Participants (N = 24)	
	Galway University Hospital	University Hospital Limerick
Total Participant Number at Each Hospital Site	13 (54.2)	11 (45.8)
Gender		
Female	9 (37.5)	8 (33.3)
Male	4 (16.7)	3 (12.5)
Country where Medical Degree was Obtained		
Ireland	10 (41.6)	9 (37.5)
Pakistan	1 (4.2)	1 (4.2)
Slovakia	0	1 (4.2)
Sudan	2 (8.3)	0
Hospital Profession		
Paediatric Consultant	4 (16.7)	3 (12.5)
Paediatric HST Program Trainee	3 (12.5)	2 (8.3)
Paediatric BST Program Trainee	3 (12.5)	3 (12.5)
Paediatric NCHD in a Non-Structured RCPI Program	3 (12.5)	3 (12.5)
Paediatric Work/Training Experience (Years)		
≤ 3	3 (12.5)	3 (12.5)
4–9	6 (25.0)	3 (12.5)
≥ 10	4 (16.7)	5 (20.8)

Abbreviations: HST, Higher Specialist Training; BST, Basic Specialist Training; NCHD, Non-Consultant Hospital Doctor

### Enablers for paediatric trainees to achieve their required procedural competencies

Participant themes on enablers that make a paediatric CBME training program successful for achieving the required procedural competencies are presented in Table 2. The theme with the highest support was have “more protected training time” (n = 16, 66.7%). This was particularly stressed by NCHDs, 10 out of 11 (90.9%) BST and HST program trainees:

Having more time where you practice the skill and get a chance to perform the procedure, the more beneficial it is.

**Table 2:** Participant Themes on Enablers that Make a Paediatric CBME Training Program Successful for Achieving the Required Procedural Competencies

		Number of Participants whose Comments Related to this Theme					
**Participant Profession**	**Number of Participants**	**Standardized curriculum**	**Dedicated resources and use of various teaching pedagogies**	**Skilled Trainers**	**Routine assessments with constructive feedback**	**More protected training time**	**Routine feedback to improve the training program**
Paediatric Consultant	7	6	5	5	4	3	1
Paediatric HST Program Trainee	5	3	2	1	3	5	0
Paediatric BST Program Trainee	6	4	3	4	4	5	2
Paediatric NCHD in a Non-Structured RCPI Program	6	2	4	2	4	3	0

Abbreviations: CBME, Competency Based Medical Education; HST, Higher Specialist Training; BST, Basic Specialist Training; NCHD, Non-Consultant Hospital Doctor

The next most common enablers were having a “standardized curriculum” (n = 15, 62.5%), “routine assessments with constructive feedback” (n = 15, 62.5%), and “dedicated resources and use of various teaching pedagogies” (n = 14, 53.4%). Dedicated resources included a simulation training facility or tools, while teaching pedagogies mentioned included bedside learning and accommodations for trainees with special needs. The theme with the lowest participant reporting was “routine feedback to improve the training program” (n = 3, 12.5%).

### Feasibility of achieving the required procedural competencies

Participant themes on whether it is feasible to achieve the required procedural competencies in the paediatric CBME training program are presented in Table 3. Most study participants agreed it is feasible to achieve the required procedural competencies in the paediatric CBME training program (n = 15, 62.5%). The theme with the highest support was “yes, there is sufficient clinical opportunities” (n = 18, 75%), which is highlighted in a participant’s quote:

I think there should be no reason why you couldn’t, there is more than enough time.

**Table 3:** Participant Perspectives and Themes on whether it is Feasible to Achieve the Required Procedural Competencies in a Paediatric CBME Training Program

			Number of Participants whose Comments Related to this Theme			
**Participant Profession**	**Number of Participants**	**Feasibility—Yes, No, or Both Yes and No**	**Yes, there is sufficient clinical opportunities**	**No, there is inconsistent clinical training and exposure**	**Yes, but more standardization in training and teaching are needed**	**Yes, but a variety of teaching approaches are needed**
Paediatric Consultant	7	Yes = 6 No = 0 Both = 1	4	1	2	4
Paediatric HST Program Trainee	5	Yes = 2 No = 0 Both = 3	4	3	0	1
Paediatric BST Program Trainee	6	Yes = 4 No = 0 Both = 2	5	2	1	0
Paediatric NCHD in a Non-Structured RCPI Program	6	Yes = 3 No = 0 Both = 3	5	3	1	1

Abbreviations: CBME, Competency Based Medical Education; HST, Higher Specialist Training; BST, Basic Specialist Training; NCHD, Non-Consultant Hospital Doctor

Conversely, 9 participants (37.5%) reported it is both feasible and not feasible to achieve the required procedural competencies in the paediatric CBME training program. Their negation of the program’s feasibility was captured in the theme “no, there is inconsistent clinical training and exposure.” For example, a participant reported:

It can be dependent on where you’re working, in certain jobs you may not be exposed to things. You can end-up getting caught into certain specialties depending on the hospital you’re in.

### Procedural competencies that are difficult to achieve and barriers to completing them

Procedural competencies identified as difficult to achieve and themes concerning barriers to completing them in the RCPI’s paediatric CBME training program are listed in Table 4. When asked about difficult procedures to achieve, six were identified. Lumbar puncture (LP) received the highest number of participants stating it was challenging to complete (n = 18, 75%). This was followed by line insertions, intubation, emergency procedures, catheterization, and suturing and casting. The order of most to least common themes concerning barriers to achieving procedural competencies in the paediatric CBME training program is: “limited clinical exposure” (i.e., certain procedures are not performed as often, too many trainees, and site variability—large versus small site patient loads; n = 19, 79.2%), “limited number of trainers and protected supervision time” (n = 13, 54.2%), “simulation and diverse teaching pedagogies required” (n = 5, 20.8%), and “trainee confidence, knowledge, and communication” (n = 2, 8.3%). For instance, one participant stated:

You don’t get too much of a turn even for the simple procedures like a lumbar puncture. It depends, if you’re working in a big unit maybe you get exposed to some procedures. If it comes, there will be maybe one or two chances, you have too many trainees, and you may not get a chance.

**Table 4:** Procedural Competencies Identified as Difficult to Achieve and Themes Concerning the Barriers to completing them in a Paediatric CBME Training Program

Procedural Competencies Reported as Difficult to Achieve				Number (%) of Participants (N = 24)	
Lumbar Puncture				18 (75.0)	
Line Insertion (intraosseous, PICC, arterial, umbilical, long, IV)				11 (45.8)	
Intubation				10 (41.7)	
Emergency Procedures (bone marrow aspirate, chest drain insertion, septic work-up, pleuritic/ascitic tap, hemodilution, NG-tube insertion, ABG collection, cardiac arrest work-up)				9 (37.5)	
Catherization (Umbilical or Urinary)				8 (33.3)	
Suturing or Casting				3 (12.5)	
Themes on Barriers to Achieving Procedural Competencies					
Number of Participants whose Comments Related to this Theme					
Participant Profession	Number of Participants	Simulation and Diverse Teaching Pedagogies Required	Limited Clinical Exposure	Trainee Confidence, Knowledge, and Communication	Limited Number of Trainers and Protected Supervision Time
Paediatric Consultant	7	2	6	1	4
Paediatric HST Program Trainee	5	1	3	1	3
Paediatric BST Program Trainee	6	2	4	0	4
Paediatric NCHD in a Non-Structured RCPI Program	6	0	6	0	2

Abbreviations: CBME, Competency Based Medical Education; PICC, Peripherally Inserted Central Catheter; IV Line, Intravenous Line; NG-Tube, Nasogastric Tube; ABG, Arterial Blood Gas; HST, Higher Specialist Training; BST, Basic Specialist Training; NCHD, Non-Consultant Hospital Doctor

### Simulation resources used and perspectives on the role of SBME to support trainees achieve their procedural competencies

Five simulation-based resources were identified as supporting trainees’ procedural competency training (Table 5). Four categories of resources were identified that involved the use of manikins, while another class of simulation-based resources mentioned included human anatomical specimens. Three participants reported there were no simulation tools used in supporting trainees achieve their procedural competencies. All four themes collected from the cohort suggest there is a role for SBME to support trainees achieve their procedural competencies. “Yes, it improves trainee confidence, experience, and expertise” (i.e., through better preparation and revision) had the highest reporting (n = 14, 58.3%) and is highlighted in one participant’s comment:

I think there is a role for paediatric simulation to teach these competencies, to help the BSTs and HSTs particularly with a skillset they may not get too much exposure to… Allows trainees to get up to a level of proficiency and mastery that is achievable… It is demonstrable that it can be translated into the clinical practice… Allows for an element of experience.

**Table 5:** Simulation Based Resources Used and Themes on whether there is a Role for SBME to Support Trainees achieve their Procedural Competencies in a Paediatric CBME Training Program

Simulation Based Resources Used				Number (%) of Participants (N = 24)	
Simulation Based Training Courses (PALS, APLS, BLS, Neonatal Day, Annual Paediatric Workshop, Neonatal Resuscitation Program, International Conferences) with Manikins				9 (37.5)	
Institution/Department (Hospital Wards and Emergency Department) Specific Simulation Labs with Manikins				8 (33.3)	
Manikins - Not Specified				8 (33.3)	
Royal College of Physicians of Ireland National Simulation Based Training Labs with Manikins				4 (16.7)	
Institution/Department Specific Simulation Training Using Human Anatomical Specimens (i.e., Umbilical Cord)				2 (8.3)	
None/Not Sure				3 (12.5)	
Themes on if there is a Role for SBME to Support Trainees Achieve their Procedural Competencies					
Number of Participants whose Comments Related to this Theme					
Participant Profession	Number of Participants	Yes, it allows for improved knowledge translation	Yes, it increases patient safety and procedural success	Yes, it improves trainee confidence, experience, and expertise	Yes, it increases trainee exposure to procedures
Paediatric Consultant	7	2	1	3	2
Paediatric HST Program Trainee	5	1	1	3	3
Paediatric BST Program Trainee	6	1	1	4	1
Paediatric NCHD in a Non-Structured RCPI Program	6	0	3	4	1

Abbreviations: SBME, Simulation Based Medical Education; CBME, Competency Based Medical Education; PALS, Paediatric Advanced Life Support; APLS, Advanced Paediatric Life Support; BLS, Basic Life Support; HST, Higher Specialist Training; BST, Basic Specialist Training; NCHD, Non-Consultant Hospital Doctor

This theme was followed by “yes, it increases trainee exposure to procedures” (n = 7, 29.2%), “yes, it increases patient safety and procedural success” (n = 6, 25%), and “yes, it allows for improved knowledge translation with trainer feedback” (n = 4, 16.7%).

## Discussion

To our knowledge, this is the first study to record the perspectives of multiple paediatric clinician stakeholders (consultants, trainees in a training program, and NCHDs in a non-structured RCPI program) on enablers and barriers for trainees to achieve their procedural competencies. Moreover, our study is the first to collect and examine paediatric consultants and trainee physicians’ thoughts on the role of SBME in assisting the learning as well as attainment of procedural competencies in a CBME training program. Finally, from a national (Ireland) perspective, this is the first study to assess the feasibility for trainees to attain their required procedural competencies in a national RCPI training program.

Many of the themes reported in our study regarding enablers that make a paediatric training program successful in allowing trainees achieve their required procedural competencies are applicable to other CBME curriculums in medicine and surgery. Perhaps the most informative themes on this topic were having protected training time, routine assessments, and a standardized curriculum. Teaching procedural skills with routine practice allows learners to effectively achieve as well as maintain a competency [15]. Moreover, a standardized training program with clear objectives creates safer and more competent physicians as well as reduces learner anxiety [16]. However, in contrast to this recommendation, there is growing interest for trainees to participate in self-directed learning (SDL) [17, 18]. Self-directed learning is considered an important component of a physician’s professional identity as it strengthens lifelong scholarship [18]. Although several studies have supported SDL as an effective teaching pedagogy, trainees have voiced their concerns with this approach and report a need for faculty guidance [18–21]. Researchers have also advised some degree of supervision should be maintained during training as trainer feedback along with trainee reflection are pillars of a CBME program [22, 23].

In addition to reducing protected time for learning and practicing procedures, SDL makes the standardization of skills difficult to achieve. This is because it reduces the amount of time a trainee can allocate to learn a required procedure versus perform other competing demands, such as balancing patient care and maintaining personal wellbeing [18, 24]. Moreover, SDL creates a learning environment that promotes unclear procedural training and outcome measures, subjectivity in certifying individual skills, and hierarchical pressures that influence training opportunities and evaluation [24]. All of these factors negatively impact the delivery of a standardized curriculum that has clear goals and instructions, which was recommended by our study participants.

Having routine assessments when acquiring a procedural skill was supported by most of our study participants as it allows for increased time to practice performing a procedure, reinforce learning, opportunities to correct errors, and receive constructive feedback based on observations made by trainers [15]. Immediate feedback and correcting an error has been shown to reduce the risk of a skill being performed incorrectly and allows for long-term memory retention [15]. Furthermore, it is recommended that trainees have the opportunity to ask questions at the end of a competency-based skill teaching session [15]. A contributing factor to a lack of assessments might be trainer feedback fatigue. This occurs when an instructor becomes overburdened by the number of assessments they are required to complete in a CBME training program. As a result, they are not completed in a timely manner or provide limited beneficial feedback [16]. A solution to trainer feedback fatigue is optimizing the type as well as length of assessments performed [16].

Most participants in our study (62.5%) reported it is feasible to achieve the required procedural competencies in the current paediatric CBME program. This is important as studies have shown that procedural skills learned during a training program are the most important attributes to help physicians in their career [25]. Conversely, roughly one-third of our study participants (37.5%) stated it is not entirely feasible to achieve the required procedural competencies in the current paediatric CBME program. There are several reasons that may explain this, such as the increasing number of procedural skills performed by non-physician clinicians due to the growth in ancillary healthcare services [26]. For example, phlebotomy and intravenous access teams are now performing procedures traditionally conducted by physician trainees. Other procedures such as providing injections, bladder catheterization, and arterial puncture are now more commonly performed by nurses [26].

Three quarters of our study participants described difficulty achieving procedural competency in performing a lumbar puncture (LP). Learning this procedure correctly is important as a traumatic LP is associated with increased treatment expenditures, unnecessary antibiotic use, and distress for the patient [27]. Unfortunately, only 24–54% of paediatric trainee attempts at performing LPs are successful [27]. The overall barrier to achieving procedural competencies identified in our study was limited clinical exposure. However, it is not uncommon for medical and surgical training programs to have inconsistencies in learning how to perform a procedural skill. This is mainly due to a limited number of available opportunities rather than having a structured curriculum [28]. Nevertheless, establishing a standardized approach for procedural skill attainment is possible and may be achieved through SBME [27]. In a recent study, participants who received SBME training were found to perform LPs at the equivalent or greater level than more senior paediatric trainees. Moreover, this competency was retained and translated into the clinical setting [27]. Although promising, further research examining the effectiveness of SBME precision teaching to train procedural skill fluency is warranted.

Several simulation tools and skill-based training programs were identified in our study as supports for trainees to learn the required procedural competencies. Moreover, SBME was recommended by all participants as an effective teaching pedagogy to support trainees achieve their procedural competencies. Using SBME has been shown to adequately prepare trainees before they perform procedures on patients [29]. Furthermore, simulation allows trainees to gain procedural experience without depending on clinical patient encounters [26]. The use of SBME tools have also been associated with improved patient care and safety. It is for these reasons, the use of simulation is advocated by the ACGME in the USA [30]. Unlike the traditional method of using logbooks to document and monitor the progress of procedural competencies, SBME allows for direct trainee observation and trainer feedback [31].

### Limitations

Our study has several limitations that include it being conducted with paediatric clinicians from only two academic tertiary hospitals. Nevertheless, these healthcare institutions are based in two of Ireland’s largest cities. Second, participant responses were subjective and dependent on the paediatric clinician’s (consultant, trainee, or NCHD in a non-structured RCPI program) understanding of what constitutes achieving a procedural competency. However, there is no standardized approach or measurement tool to assess whether a procedural competency has been achieved. Finally, the primary author’s (WUK) own understanding and experience with procedural training may have influenced the interpretation of the study’s findings. To reduce this potential bias, analyses and interpretations of the study’s findings were discussed among several authors until a consensus was established. **Conclusion** Overall, the achievement of procedural skills is a fundamental aspect of a paediatric CBME training program. Our study identified a number of enablers to facilitate this as well as barriers that hinder this goal. The use of SBME was unanimously recommended by our cohort as an effective tool to assist in the achievement of procedural competencies and most participants reported it feasible to attain the required procedural skills in the current paediatric CBME program. Further research is needed to obtain the perspectives of paediatric clinicians from different regions and countries to ensure our findings are generalizable to other CBME training programs. Longitudinal cohort and cost-effectiveness studies are also warranted to determine the feasibility of implementing our study participants’ recommendations nationwide as well as in other country’s CBME training programs. Finally, research on how to effectively evaluate the achievement of procedural competencies is necessary given that attaining these skills can have a significant impact on the quality of trainers, trainees, and ultimately patient care.

## Data Availability

The datasets used and analyzed during this study are available from the corresponding author on reasonable request.

## List of abbreviations

CBME: Competency-based medical education
WHO: World Health Organization
ACGME: Accreditation Council for Graduate Medical Education
USA: United States of America
SBME: Simulation-based medical education
RCPI: Royal College of Physicians of Ireland
GUH: Galway University Hospital
UHL: University Hospital Limerick
HST: Higher specialist training
BST: Basic specialist training
NCHD: Non-consultant hospital doctor
IV Line: Intravenous Line
NG-Tube: Nasogastric Tube
ABG: Arterial Blood Gas
PALS: Paediatric Advanced Life Support
APLS: Advanced Paediatric Life Support
